# Surgical Planning of Sacral Nerve Stimulation Procedure in Presence of Sacral Anomalies by Using Personalized Polymeric Prototypes Obtained with Additive Manufacturing Techniques

**DOI:** 10.3390/polym12030581

**Published:** 2020-03-05

**Authors:** Inés Rubio-Pérez, Andrés Díaz Lantada

**Affiliations:** 1 Servicio de Cirugía General, Hospital Universitario La Paz, Paseo de la Castellana 261, 28046 Madrid, Spain; i.rubio@aecirujanos.es; 2 Laboratorio de Desarrollo de Productos, Departamento de Ingeniería Mecánica, ETSI Industriales, Universidad Politécnica de Madrid, c/José Gutiérrez Abascal 2, 28006 Madrid, Spain

**Keywords:** surgical planning, additive manufacturing, rapid prototyping, fused deposition modeling, laser stereolithography, sacral nerve stimulation, biomedical engineering, personalized medicine

## Abstract

Sacral nerve stimulation or sacral neuromodulation involves the implantation of a stimulating electrode lead through the sacral foramina. In patients with anatomical sacral anomalies, it can constitute a challenging procedure due to a lack of common reference points present in the normal anatomy. In this study, we present an innovative application of additive manufacturing for the planning of sacral nerve stimulation techniques and related surgical procedures in complex cases, and we verify that the use of personalized patient models may help to manage the presence of sacral anomalies. The use of two alternative additive manufacturing technologies working with thermoplastic and thermoset polymers, including fused deposition modeling as low-cost alternative and laser stereolithography as industrial gold standard, is compared in terms of viability, precision and overall production costs. They pay special attention to fidelity in terms of the bone microstructure reconstruction, which is necessary for adequately planning electrode insertion. Advantages and limitations of the alternative approaches are discussed and ideas for future developments and for solving current challenges are presented.

## 1. Introduction

Sacral nerve stimulation (SNS) or sacral neuromodulation involves the implantation of a stimulating electrode lead through the sacral foramina, which stimulates the nerve and appears to modulate colonic and urinary function locally and via central nervous activity. This has demonstrated a remarkable efficacy for the treatment of several colorectal and urinary conditions, such as urinary retention, fecal incontinence, constipation, and other bowel dysfunctions [1,2].

However, in patients with anatomical sacral anomalies, it constitutes a challenging procedure due to the lack of common reference points present in the normal anatomy, which are essential to locate the appropriate sacral foramina and perform the standardized implantation technique. These reference points are clearly seen in simple radiology (anterior and lateral projections), computed tomography (CT) images, and nuclear magnetic resonance images (MRI) and can be landmarked by simple visual inspection by surgeons during the interventions.

Current solutions for the more complex or anomalous sacral anatomies rely on intra-operative CT combined with neuronavigation, a synergistic use of advanced technologies leading to quicker and safer guidance [3]. Nevertheless, intra-operative CT navigation for spine surgery implies additional radiation exposure, both to patients—almost up to three times more—and to surgeons, who receive a not negligible dose even if the patients are exposed to almost nine times more radiation than the medical professionals [4].

In consequence, alternative or complementary solutions to simplify these procedures in presence of sacral anomalies, while minimizing radiation exposure, would be welcome. Among current trends in the use of advanced additive manufacturing techniques (AMT) in the biomedical field [5], it is important to cite the production of biological and anatomical models to support surgical training and planning tasks as well as the development of prototypes to simplify surgical procedures. Additive manufacturing technologies, typically working by adding material on a layer-by-layer fashion, enable the creation of very complex geometries (such as those from the human body) which is very adequate for the generation of biomimetic models for a wide set of purposes.

Pioneering research, linked to the production of prototypes of biological models for surgical planning tasks, includes fields as diverse as: neurology [6], traumatology [7], maxillofacial surgery [8,9], digestive tract surgery [10], or heart and cardiovascular surgery [11]. More recently, these techniques have been also applied to spinal surgery planning and to the development of support surgical guides most notably in relation to cervical and lumbar diseases and tumors, as reviewed elsewhere [12].

To summarize, surgical planning by means of additively manufactured rapid prototypes of biological structures is now common in several technologically advanced hospitals, many of which are even starting to internalize computer-aided design and conventional 3D printers, generally for planning and training applications. In any case, novel medical uses for these technologies, especially when combined with state-of-the-art medical imaging hardware and software, are being continuously developed thanks to multidisciplinary collaboration between medical professionals, engineers, and technology developers.

In this study, we present an innovative application of additive manufacturing for the planning of sacral nerve stimulation techniques and related surgical procedures. We verify that the use of personalized patient models may help to manage the presence of sacral anomalies. The use of two alternative additive manufacturing technologies, including fused deposition modeling (FDM and commonly referred to as “3D printing”) as low-cost alternative and laser stereolithography (SLA) as industrial gold standard, is compared in terms of viability, precision, and overall production costs, paying special attention to fidelity in terms of the bone microstructure reconstruction. This is necessary for adequately planning electrode lead insertion and placement. Advantages and limitations of the alternative approaches are discussed, and ideas for future developments for enhanced results and for solving current challenges are presented. Taking into account the aforementioned very recent and comprehensive review [12], which includes more than 20 different applications of additively manufactured prototypes for spinal surgery planning, none of which deals with sacral nerve stimulation, we consider this proposal as one-of-a-kind to the best of our knowledge.

Regarding the case of study selected for validating the proposed strategy, it is important to mention that it was chosen due to its complex anatomy of the sacral zone and the anticipation of technical complications during the procedure. In these complex cases, surgical solutions are very limited, and procedures such as SNS can be the last therapeutic chance for patients otherwise condemned to chronic symptoms and impaired quality of life despite their young age. The SNS procedure can be challenging for the surgeon, who usually depends only on expertise to achieve adequate electrode placement, aided only by a simple X-ray. To optimize the procedure and increase the possibilities of success, preoperative planning and knowledge of the specific anatomy are fundamental.

We consider that the described case provides a very representative practical example of translational research of the advantages of promoting collaboration among medical professionals and engineers. It also helps to put forward how rapid prototypes can help surgeons in the clinical setting and aid in successful outcomes for selected patients.

## 2. Experimental Section

### 2.1. Case Description

We selected the case of a 25-year-old male with faecal incontinence secondary to congenital malformations and previous reconstructive surgery in infancy. The patient was born with anal atresia (absence of anatomical anal complex) and other associated malformations. After various surgical interventions, the patient recovered completely, but presented passive faecal incontinence with continuous soiling, reduced rectal sensitivity, and urgency which severely affected his quality of life. The patient had visited various hospitals referred from his paediatricians to consider the possibility of an anal artificial sphincter, or any other alternative therapy, without success. Now in adulthood, the patient was referred to the specialized clinic on incontinence and pelvic floor disorders of the Colorectal Surgery Department at Hospital Universitario La Paz for expert opinion.

After revising past medical history and completing investigations, the patient was offered SNS therapy, which he accepted. The sacral malformation consisted of a dysplasia with a partial agenesis, with unilateral fusion of sacrococcygeal S4–S5 foramina and absence of coccyx, but with permeable S3 foramina that allowed for electrode insertion. The procedure was performed routinely with electrode placement in S3 foramina under local anesthetic with an intraoperative simple X-Ray (posterior-anterior and lateral projections). Electrode placement was challenging, and surgeons highlighted the importance of preoperative planning and how a better understanding of the anomalous anatomy could have improved and eased the procedure.

### 2.2. Medical Imaging and Segmentation Software

Medical imaging was performed using an Aquilion ONE™ helical CT scan with intravenous contrast (Iomeron 400 mg Iodine/mL), total DLP (mGy·cm) 853.40 (Body), with 3D post-processing reconstruction with TeraRecon, and the information was stored using DICOM (digital imaging and communications in medicine) in dcm files for subsequent processing.

3D Slicer (v.4.8), an open-source software platform for medical image informatics, image processing, and three-dimensional visualization was used for generating three-dimensional reconstructions of the biostructures of interest for the surgical planning. 3D Slicer provides an open-source alternative to other typically very expensive software for generating CAD (computer-aided design) files starting from medical images. 3D Slicer provides similar functionalities to those from commercial options and even improved versatility thanks to being developed through the contributions from an international community of scientists from a multitude of fields, including engineering and biomedicine [13,14].

Using an anonymized patient’s medical images (in compliance with national personal data protection law), and after importing the dcm file with 3D Slicer, two different segmentations were performed, taking into account that our main objective was to reconstruct the sacral complex with the anatomical anomalies of the patient’s bones. Considering that in medical imaging technologies, some overlap may appear between soft tissues (typically in the range of 100–300 Hounsfield units) and spongy bone (typically in the range of 200 to 700 Hounsfield units or HU), we decided to compare two segmentations. Segmentation A was created by selecting the range (200–2000) of the Hounsfield scale and includes density values in the range of surrounding cartilages, whose impact is discussed in Section 3. Segmentation B was generated by selecting the range (250–2000) of the Hounsfield scale for focusing on the higher densities, mainly those of bone, and checking that if the lower limit of 250 was moved up to values around 275–300HU, and then some relevant bone microstructures were lost.

Once generated, both segmentations were saved as stl (standard tessellation language or stereolithography) files for subsequent prototyping tasks.

The decision and impacts of working with two segmentations are discussed in Section 3. In any case, it is important to advance that these segmentations should be performed by professionals trained in the use of medical imaging and 3D Slicer software (which is quite intuitive) and with the overview of medical professionals familiar with the anatomical structures of interest. In the presence of anomalies, counting with medical professionals having a good knowledge of available treatments, surgical procedures, and expectable deviations from the norm is also essential for supporting technicians.

### 2.3. Manufacturing Resources and Materials

Two additive manufacturing technologies were used for generating the surgical planning models and validating a low-cost option, based on fused deposition modeling, and an industrial gold standard, using laser stereolithography, the first relevant additive manufacturing technology with industrial impact. Fused deposition modeling works thanks to a heated extruder that creates superimposed layers of thermoplastic polymer by depositing lines of molten polymer filament, while laser stereolithography works on the basis of a laser beam that selectively photo-polymerizes a liquid resin, again layer-by-layer, hence making it turn into a solid object.

In the case of fused deposition modeling a BCN3D Sigma machine (BCN 3D Technologies, c/Esteve Terradas, 1, 08860, Castelldefels, Barcelona) was used, whereas in the case of laser stereolithography we employed a SLA-3500 System by 3D Systems (3D Systems, 333 Three D Systems Circle, Rock Hill, SC 29,730 USA). PLA (poly-(lactic acid)) filament (ivory white Smartfil PLA by Smart Materials 3D, Alcalá la Real, Jaén, Spain) was used for fused deposition modeling and epoxy resin (DSM Somos^®^ WaterShed XC 11122, DSM Desotech B.V., P.O. Box 68, 3150 AB Hoek van Holland, The Netherlands) was employed for the stereolithography models.

The deposition and writing paths for both manufacturing systems were generated by working with the stl files of both segmentations and with two slicing software: Open-source Cura 3.3 by Ultimaker B.V. for the fused deposition modeling parts and 3D Lightyear by 3D Systems for the laser stereolithography objects. Special attention was paid to the placement of the segmentations in the construction platforms as the back part of the sacral structure, through which the surgical intervention is performed, should be placed in a position similar to that of the patient during the surgical procedure (prone), so that the supporting structures generated during the manufacture would affect its final surface quality as little as possible. The working platforms of both machines allowed for the manufacture in a single job of both segmentations, although the laser stereolithography machine has a larger construction envelope than the FDM one, which should be considered for larger reconstructions.

Finally, four models were obtained using the two segmentations and the two available technologies as discussed in the results section. Support structures were manually eliminated paying careful attention not to affect the inner bone microstructure during the process. The main advantages and drawbacks of the different alternatives employed are presented and discussed in the following section.

Table 1 compares the features of the additive manufacturing technologies employed for the study and details some manufacturing parameters adjusted for enhanced performance considering the size of models to be obtained. Additionally, Table 2 includes information about the materials employed. It is important to highlight that the materials employed are not implantable and do not get in contact with the patient in any stage of the planning process. The materials and obtained models are used by physicians, as in vitro models, for analyzing and planning surgery. In the case of devices used for interacting with the patients during surgery (i.e., surgical guides, supports for surgical instruments, among others), it would be necessary to employ other materials and compliance with ISO 10993, if the materials and devices contact the patient, should be considered.

Additional details regarding quality of the prototypes manufactured, manufacturing time, costs involved and applicability to surgical planning are presented in the results and discussion sections, in which future proposals are also analyzed.

### 2.4. In Vitro Surgical Planning

A demonstration surgical kit from Medtronic (Model 3550-18/042294 Lead Introducer Kit and Model 3093 Inter Stim Extended Electrode Quad Tined Lead) was used to check, in vitro, the potential use of the additively obtained models for surgical planning in relation to the sacral nerve stimulation procedure. The prototypes were placed in prone position (as is the patient in the procedure) and the lead introduction needles and dilator were used over the sacral foramina, considering the anatomical position and correct angulations needed for an adequate insertion, thus mimicking real surgery. Obtained both by FDM and SLA and with two segmentations, the use of four different prototypes helped to compare the technologies and materials employed in terms of usability and to point out the relevance of performing an adequate segmentation supported by imaging diagnostic professionals, as further described in the results and discussion sections.

## 3. Results

### 3.1. Design and Prototyping Results

**Figure 1:**
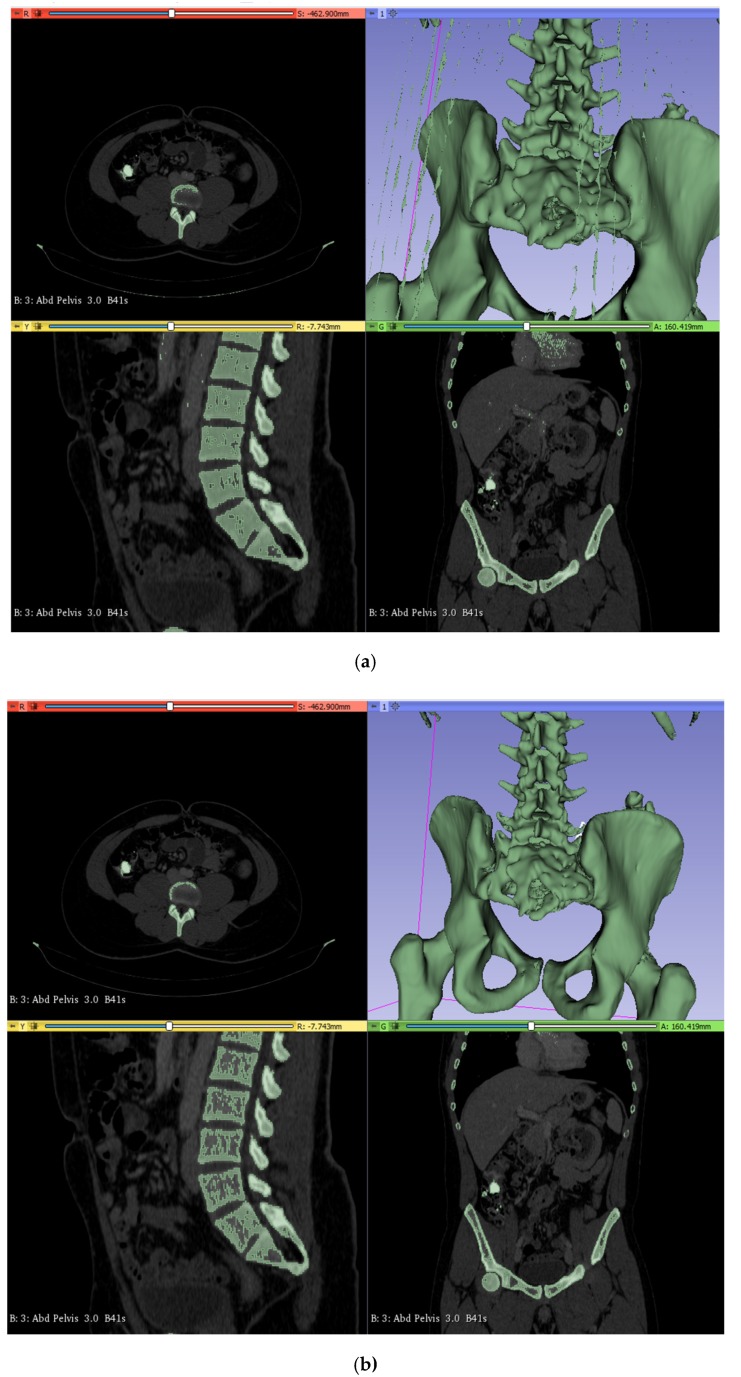
(**a**) Segmentation A, including the lower densities. (**b**) Segmentation B, focusing on the higher densities. Three-dimensional CAD volumes creation directly from the medical images stored in DICOM format and thanks to the use of 3D Slicer [13,14] open-source software.

Figure 1 shows the previously detailed segmentation, including the one created by selecting the range (200–2000) of the Hounsfield scale (Figure 1a) and the one generated by selecting the (250–2000) range (Figure 1b). The level of detail can be appreciated and is similar to the quality obtained with state-of-the-art visualization and segmentation tools, in spite of 3D Slicer being an open-source alternative to much more expensive commercial software, which makes us feel optimistic about the potential expansion of current proposal, possibly even to low- and middle-income settings.

**Figure 2:**
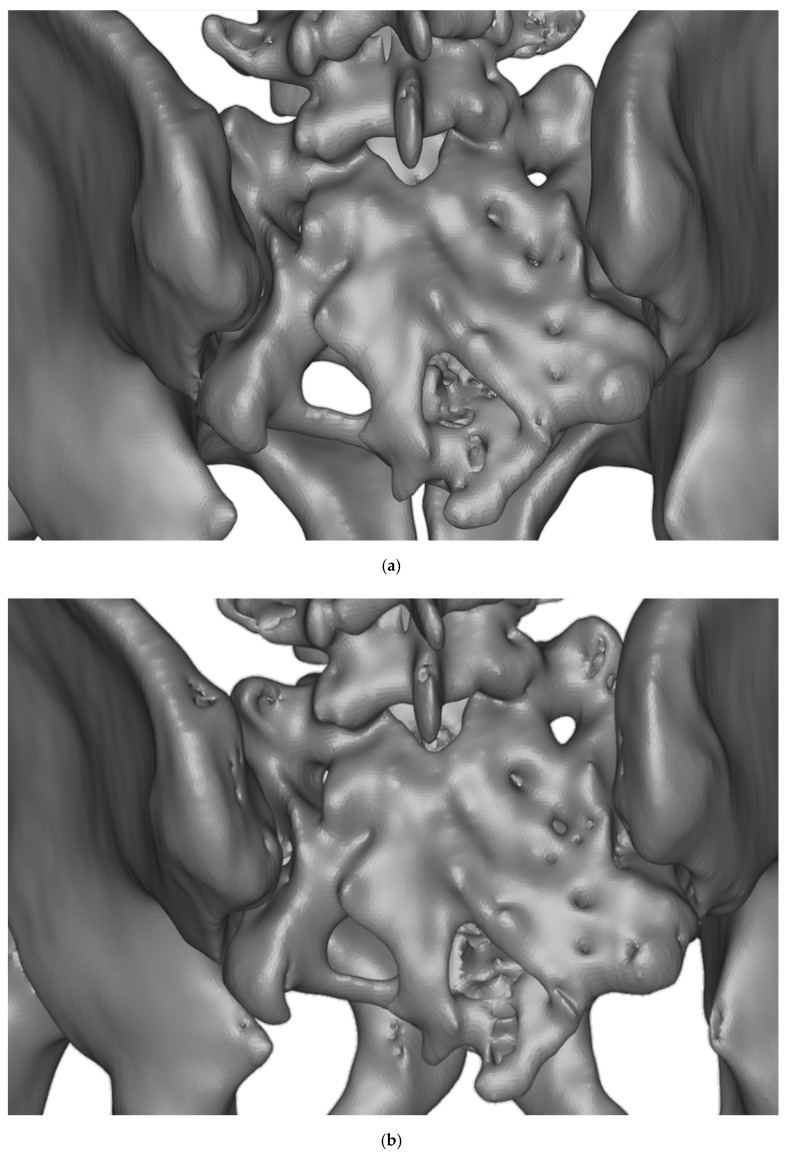
Three-dimensionally reconstructed models with differences due to the density values selected for each segmentation. The high density segmentation (**b**) provides and enhanced representation of bone microstructure than the reconstruction including lower densities (**a**), in which the presence of cartilage and surrounding soft tissues and fluids prevents the reconstruction of actual existing channels, which are relevant for the surgical procedure, especially as regarding the presence of surgical paths for inserting the electrodes for stimulation. Scale bar: 90 mm.

Three-dimensional CAD volumes are hence created directly from the medical images stored in DICOM format and can be converted into stl files, which are shown in Figure 2. It again includes the (200–2000) range and the (250–2000) range segmentations, shown in Figure 2a,b, respectively.

We would like to highlight that the high density segmentation (Figure 2b) provides and enhanced representation of bone microstructure than the reconstruction including lower densities (Figure 2a), in which the presence of cartilage and surrounding soft tissues and fluids may prevent the reconstruction of actual existing channels, which are relevant for the surgical procedure, especially as regards the presence of surgical paths for inserting the electrodes for stimulation.

In any case, we decided to continue with the manufacture of both segmentations using the available technologies. This was done in order to check the actual differences in the real surgical models, to analyze their viability, and the influence of both segmentation, printing technology, and employed materials in the final performance of the surgical models, as well as to put forward the need for fluid communication between the medical and technical professionals when developing these sort of biological models for surgical practice.

The more interesting differences between the three-dimensional reconstructions are marked in Figure 2b with surrounding ellipses, which help to make clear the improved reproduction of actually existing surgical paths for introducing and placing the electrode leads. These have been advanced by the dedicated software of the medical imaging technology and should be also present in the corresponding segmentations and prototypes.

**Figure 3:**
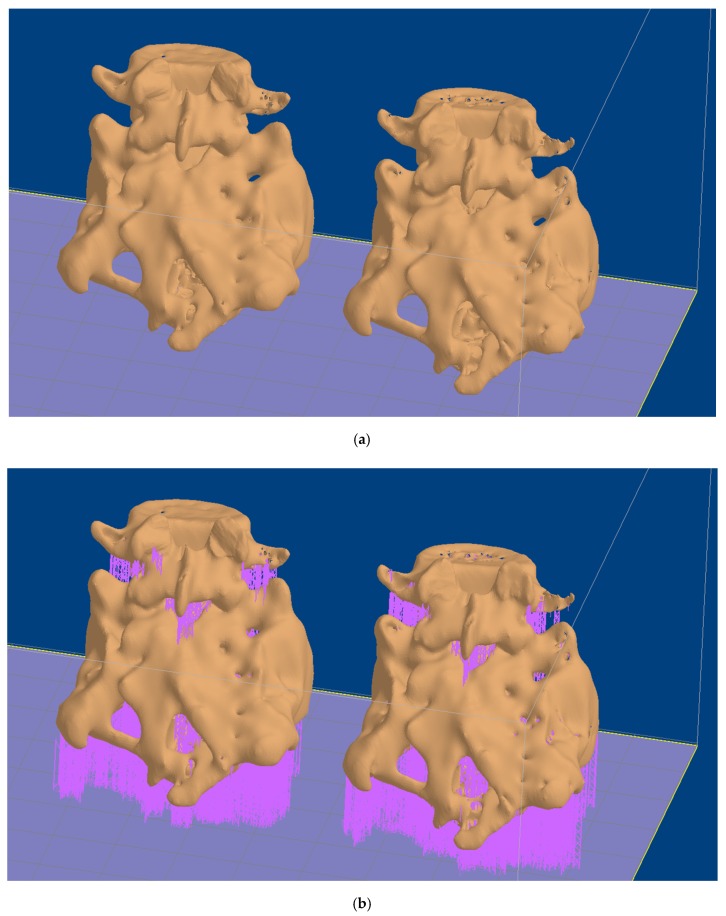
Planning of rapid prototyping process by laser stereolithography: (**a**) Positioning of alternatives in the construction platform. (**b**) Generated support structures. Scale bar: 90 mm.

After checking the interest of the segmentations and 3D CAD reconstructions with the team of surgeons, especially regarding those from Figure 1b and Figure 2b, the stl files were loaded with the two mentioned slicing software corresponding to both manufacturing alternatives (Cura for the FDM process and 3D Lightyear for the laser stereolithography procedure).The 3D models were placed in the most appropriate printing position, considering that the functional zone of the surgical models should include as fewer supporting structures as possible. By means of example, Figure 3 shows the planning of the rapid prototyping process by laser stereolithography, including the positioning of alternatives in the construction platform (Figure 3a) and the generated support structures (Figure 3b).

Once manufacturability was checked layer by layer by reviewing the 3D printing and laser polymerization trajectories, generated by the slicing software, manufacturing was accomplished and both alternatives (fused deposition modeling and laser stereolithography) provided complete physical prototypes without relevant manufacturing errors or missing zones.

**Figure 4:**
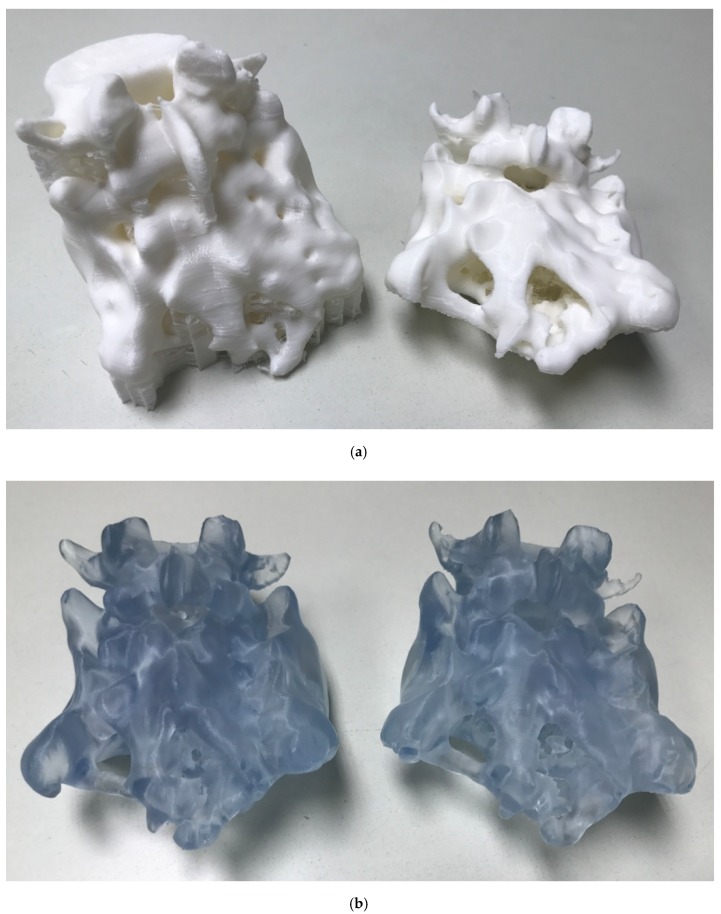
Prototyping results: (**a**) Surgical planning models obtained by fused deposition modeling (FDM). (**b**) Surgical planning models manufactured by laser stereolithography (SLA). The ones focusing on the higher densities (right images segmentation (B) provide more detailed bone structures, with the actual existing channels for planning the access of the electrodes for stimulation, than those including also the lower densities (left images: segmentation (A). Scale bars: 90 mm.

Figure 4 shows the prototyping results, including the surgical planning models obtained by fused deposition modeling (Figure 4a) and the surgical planning models manufactured by laser stereolithography (Figure 4b). Figure 4a shows a model still with the supporting structure (left image) and a model after manual elimination of supports (right image). From direct visual inspection, the prototypes based on segmentation B (right images) provide more detailed bone structures with the actual existing channels for planning the access of the electrodes for nerve stimulation than those also including the lower density values and made following segmentation A (left images).

### 3.2. Evaluation of Alternative Design Options and Manufacturing Technologies

Table 3 and Table 4 provide a quantitative evaluation of design (segmentations A and B) and rapid prototyping alternatives (fused deposition modeling and laser stereolithography).

Table 3 includes details on part volumes and areas of support structures, as measured with the CAD software, and presents actual masses of parts and supports measured upon the actually manufactured prototypes, together with manufacturing times for both alternatives.

It can be appreciated that laser stereolithography models are denser and that “segmentation B” requires additional support structures, due to a better reconstruction of inner bone microstructure, holes and features. Besides, the values from Table 3, together with additional data included in Table 4 regarding costs of materials and machines amortizations, let us compare both manufacturing options in terms of overall costs.

In summary, laser stereolithography models may be up to around 4.5 times higher in cost, especially when using high quality industrial systems as the SLA-3500 by 3D Systems, than when resorting to low-cost fused deposition modeling processes. However, the enhanced speed (more than two times faster) and the higher precision of laser stereolithography, when compared to FDM (see Table 1 and Table 2), makes us propose stereolithography-based models as the most adequate alternative for precise and fast surgical planning.

Although both technologies are remarkable in terms of X/Y/Z resolution, the laser-based one polymerizes lines of 0.24 mm, while the FDM works with printed lines of 0.4 mm. In consequence, the minimum achievable thickness and details are finer in the case of laser stereolithography. In the prototypes obtained by laser stereolithography, pores and channels of around 300 μm are commonly appreciated, while in the FDM prototypes, similar geometries tend to appear closed due to the impact of the thicker filament, as compared with the laser beam. Besides, SLA prototypes, being semi-transparent, allow for enhanced visualization during planning. In any case, FDM is also interesting, as the fused deposition modeling prototypes may serve as teaching models for anatomical practices.

**Figure 5:**
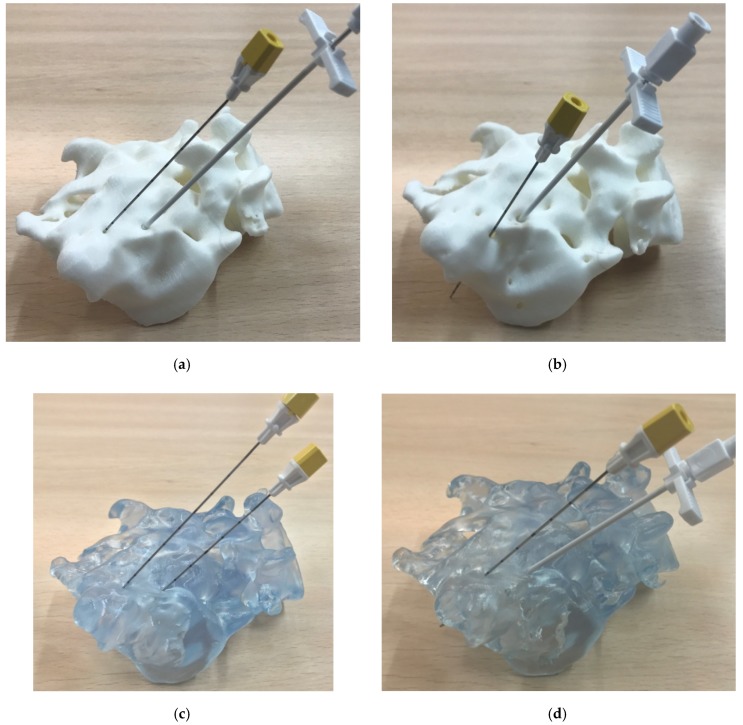
Surgical planning using the generated models: Geometrical differences and validation of the most adequate segmentation and technique for these interventions: (**a**,**b**) FDM prototypes. (**c**,**d**) Laser stereolithography prototypes. (**a**–**c**) Segmentation A. (**c**,**d**) Segmentation B. The denser segmentation (A) did not reconstruct the finer details of the bone structure and did not allow the surgical tools to enter the desired inner zones for electrode placement.

The enhanced quality of the laser stereolithography models and the adequacy of segmentation B can be further understood by visual inspection of Figure 5, in which the surgical planning is shown using the generated models or physical prototypes and the aforementioned demonstration surgical kit from Medtronic. In such figure, the upper row shows the FDM prototypes and the lower row the laser stereolithography ones, while segmentations are divided with the A ones to the left and the B ones to the right.

The denser segmentations (segmentation A) did not reconstruct the finer details of the bone structure and did not allow the surgical tools to enter the desired inner zones for electrode placement. This highlights the need for taking account of the synergies between segmentation, prototyping technology, and material uses. It also insists on the relevance of intimate collaboration among medical professionals, designers, and manufacturers during the creation of models for surgical training, as the adequate segmentations have to be selected according to the anatomical details highlighted by the medical professionals.

## 4. Discussion

We consider that prototyping results are promising, although we also envision some potential improvements for the future. For instance, the use of washable support materials, thanks to the possibility of using dual fused deposition modeling and hence printing with two materials, typically one water-resistant for the structure and one water-soluble for the supports, may help to minimize post-processing tasks in the FDM alternative and promote an easier elimination of supports and higher fidelity.

Similar prototypes can be implemented in the form of work-benches, not only for patient-specific surgical planning procedures, but also as surgical training benches made of rapid prototypes mimicking and integrating different body structures and using varied materials and manufacturing processes (i.e., additive rapid prototyping, rapid form copying in PDMS molds, injection of thermoplastics and elastomers) for obtaining a realistic biomechanical response.

Regarding the impact of these surgical procedures and the potential benefits from technology-assisted planning, it is important to mention that the SNS technique has been now available for over 30 years and has consolidated widely as a valid option for the treatment of various urinary and defecatory dysfunctions. Patient follow-up shows improved outcomes dependent on adequate electrode placement and programming, so importance has been given to a standardized technique for electrode placement. Lately, a European consensus of experts described the optimal technique step by step to try and optimize outcomes and avoid inter-surgeon variability in practice [15].

Some other innovative strategies have been developed in the field of sacral neuromodulation, always aiming to facilitate technical aspects of electrode insertion or optimize outcomes. Recently, as previously mentioned, neuronavigation and CT-guided procedures has been successful in aiding electrode insertion [3], even with the development of experimental animal models to try and optimize standard techniques [16]. However, possible limitations of this technique are the availability of a CT-scan suite for the intervention, the difficulty in strictly defining the insertion zone with a patient in prone position and awake, who can move and be uncomfortable if kept still for a prolonged period of time, and the high radiation of CT compared with simple X-ray. Also, the SNS technique implies the implant of a prosthetic material (electrode) and should be performed under sterile conditions. Unless the CT-scan is integrated in a surgical theatre, radiology suites may not have the appropriate conditions such as air filters and positive pressure that are present in theatres to ensure an optimal environment.

In our opinion based on the presented results, prototyping of anatomical models can aid the surgeon in planning the intervention, by actually manipulating a 3D model of the structure, which is intuitively better than only seeing the 3D reconstruction on screen. Our proposal sums to pioneering examples of surgical planning aided by 3D image reconstruction and patient avatars, either supported by physical prototypes or not, which have proven successful for bone tumor surgeries [17], for craniofacial surgeries [18], for orthognathic surgery [19], for kidney surgeries [20], for general surgery [21], and for heart surgery [22], to cite just some relevant studies.

Moreover, the possibility of practicing the electrode insertion procedure with the model and evaluating the optimal insertion angles can be an excellent way of training ‘ex-vivo’ and avoiding patient discomfort, reducing the number of attempts and insertion and operating time of the procedure, while increasing the possibilities of success. These models can also be helpful for educational purposes in workshops and even wet labs to train surgeons in SNS placement and achieve a better understanding of the surgical anatomy.

Sharing of these sorts of human models for surgical planning and training through open-source biomedical engineering platforms and collaborative design environments may help to promote the medical impact of these innovative procedures [23] and to progress towards medical device personalization [24]. Exploring connections to virtual surgical planning [25] may open additional continuation lines, especially for patients with malformations, in which virtual planning plays a more relevant role [26].

## 5. Conclusions

We have presented an innovative application of polymeric additive manufacturing for the planning of sacral nerve stimulation techniques and related surgical procedures and verified that the use of personalized patient models may help to manage the presence of sacral anomalies. The use of two alternative additive manufacturing technologies (fused deposition modeling of thermoplastic polymers and laser stereolithography of thermoset resins) has been compared in terms of viability, precision and overall production costs. The laser stereolithography-generated prototypes, in spite of resulting more expensive, are more rapidly obtained and provide enhanced fidelity.

## Figures and Tables

**Table 1:** Characteristics of the additive manufacturing technologies according to the manufacturers’ data sheets and manufacturing parameters employed for this study.

Characteristics and Manufacturing Parameters	Fused Deposition Modeling (FDM)	Laser Stereolithography (SLA)
Company and model	BCN 3D, Sigma R19	3D Systems, SLA-3500
Maximum build envelope	210 × 297 × 210 mm^3^	350 × 350 × 400 mm^3^
Nozzle diameter/laser width	0.4 mm	0.2–0.3 mm
Positioning resolution (X/Y/Z)	1.25 µm/1.25 µm/1 µm	5 µm/5 µm/1 µm
Selected layer thickness	0.15 mm	0.15 mm
Printed filament line width/polymerized line width	0.4 mm	0.24 mm
Laser source	-	Solid State Nd:YVO4, 354.7 nm

**Table 2:** Characteristics of the materials employed for this study according to materials data sheets.

Characteristics	FDM-PLA	SLA-Epoxy
Polymer employed	Thermoplastic PLA	Thermoset epoxy resin
Manufacturer	Smart Materials 3D	DSM Desotech
Commercial name	Smartfil PLA	Somos^®^ Water Shed XC 11122
Colour	Ivory white (opaque)	Optically clear, nearly colorless
Density	1.24 kg/m^3^	1.12 kg/m^3^
Tensile strength	53 MPa	47.1–53.6 MPa
Tensile modulus	3.6 GPa	2.65–2.88 GPa
Glass transition	55–60 °C	39–46 °C
Print temperature	220 °C (+/−20 °C)	Standard conditions
Filament diameter	2.85 mm	-
Printed diameter	0.4 mm	-

**Table 3:** Evaluation of design and prototyping alternatives: Quantitative data obtained from CAD models and from the actual manufactured prototypes with the different segmentations, materials, and technologies employed for present case of study.

	CAD Models		FDM Using PLA as Material		Laser Stereolithography Using Epoxy Resin	
	Part Volume (mm^3^)	Area of Supports (mm^2^)	Mass of Part and Supports (g)	Mass of Final Parts & Mass of Supports (g)	Mass of Part and Supports (g)	Mass of Final Parts & Mass of Supports (g)
**Segmentation A (200–2000HU)**	185	28,300	107	90 & 17	231	209 & 22
**Segmentation B (250–2000HU)**	133	38,900	102	83 & 19	192	164 & 28
**Manufacturing time**	-		23 h		10 h and 50 min.	

**Table 4:** Cost evaluation regarding the different alternatives: Based on the actual quantities and cost of materials used, on the different manufacturing times and on real machine amortizations.

Technologies and Models		Mass (kg)	Cost (€/kg)	Time (h)	Amortization (€/Hour)	Total (€) [M·C+T·A]
**FDM**	**Segmentation A (200–2000HU)**	0.107	12	11.5	6	70.3
	**Segmentation B (250–2000HU)**	0.102	12	11.5	6	70.2
**Laser stereo-lithography**	**Segmentation A (200–2000HU)**	0.231	62	5.4	58	327.5
	**Segmentation B (250–2000HU)**	0.192	62	5.4	58	325.1
